# BushenHuoxue Recipe for the Treatment of Prethrombotic State of ACA-Positive Recurrent Miscarriage via the Regulation of the PI3K-AKT Signaling Pathway

**DOI:** 10.1155/2022/2385534

**Published:** 2022-02-14

**Authors:** Xuan Yang, Shulan Su, Qingling Ren, Lijing Liu, Jiashang Li, Wen Zhang, Ke Cai, Zhuo Xu, Xin Pan

**Affiliations:** ^1^Affiliated Hospital of Nanjing University of Chinese Medicine, Nanjing 210004, China; ^2^Anhui Province Hospital of Integrated Traditional Chinese and Western Medicine, Hefei 230031, China; ^3^Jiangsu Key Laboratory for High Technology Research of TCM Formulae and Jiangsu Collaborative Innovation Center of Chinese Medicinal Resources Industrialization, Nanjing University of Chinese Medicine, Nanjing 210023, China

## Abstract

**Background:**

Although the Bushen Huoxue (BSHX) recipe is commonly used for the effective treatment of the prethrombotic state of recurrent abortions, its mechanism of action is unclear. In this article, we investigated the therapeutic effects of BSHX on anti-cardiolipin antibody (ACA) positive recurrent miscarriage mice and the molecular mechanism involved in the treatment of the prethrombotic state of ACA-positive recurrent miscarriages based on the PI3K-Akt signaling pathway, to provide a scientific basis for clinical practice.

**Methods:**

An ACA-positive recurrent miscarriage mouse model and normal pregnancy mouse model were adopted in this experiment. Seventy CBA/J female mice were induced to establish the ACA-positive recurrent model; the mice were mated with DBA/2 male mice. Of these mice, 50 became pregnant, which were randomly divided into a BSHX high-dose group (BH, 2.52 g/kg), BSHX medium-dose group (BM, 1.26 g/kg), BSHX low-dose group (BL, 0.63 g/kg), model group (M, distilled water), and an aspirin enteric-coated tablet group; each group had 10 mice. In addition, 16 CBA/J female mice were induced to establish the normal pregnant mouse model; the mice were mated with BALB/C male mice. Of these mice, 10 became pregnant, which were used as the blank control group (C) and received distilled water by gavage. Stillbirth and abortion rates were recorded for each group, and the uterine tissue, urine, and serum were collected. The serum expression levels of ACA, interleukin-6 (IL-6), progesterone ,estradiol, and endometrial histological changes were compared between the groups. Metabolomics was performed on the urine and uterine tissues of both groups using UHPLC-QTOF/MS, and the expression levels of PI3K, *p*-PI3K, AKT, and *p*-AKT proteins in the uterine tissues were detected using Western blot.

**Results:**

Compared with the model pregnancy group, the BSHX high-dose group, BSHX medium-dose group, and BSHX low-dose group all had a lower absorption rate of mouse embryos, improved uterine histopathological morphology, significantly reduced serum levels of ACA and IL-6, increased serum levels of progesterone and estradiol, and significantly upregulated uterine levels of *p*-AKT, PI3K, and *p*-PI3K proteins. The metabolomic results showed that the metabolic levels in the urine and uterine tissues were significantly altered in the mouse model of ACA-positive recurrent abortion. The results also suggested that the pathogenesis of ACA-positive recurrent abortion may be associated with metabolic pathways, such as pentose, glucuronide, lysine degradation, and steroid hormone biosynthesis.

**Conclusion:**

The BSHX recipe improved the uterine histopathological morphology of pregnant mice and promoted vascular formation in uterine tissues. The mechanisms involved the reduction in serum ACA and IL-6 levels, the increment in serumprogesterone and estradiol levels, the upregulation of the levels of *p*-AKT, PI3K, and *p*-PI3K proteins, and the activation of the PI3K-Akt signaling pathway. These data will be useful for effective drug research and development.

## 1. Introduction

Recurrent spontaneous abortion (RSA), also called habitual abortion, is defined as three or more consecutive spontaneous abortions before 28 weeks of gestation [1]. RSA has a complex pathophysiology, and its etiology is primarily related to genetics, immune system, endocrine system, prethrombotic state (PTS), and infections [2].

PTS, also known as thrombophilia, is a pathological state that predisposes to thrombosis. It was formerly called a hypercoagulable state, and the pathological process involves dysfunction of the anti-coagulation, coagulation, and fibrinolytic systems due to a combination of internal and external factors. PTS during pregnancy affects the placenta as well, thereby predisposing to local microthrombi and even placental infarction. Placental microthrombi and/or infarction decrease the placental blood supply to the placenta, which causes ischemia, hypoxia, and cardiovascular risk to the embryo or fetus, eventually leading to miscarriage due to embryonic or fetal failure [3–5]. PTS can be classified as hereditary or acquired, with the former primarily caused by genetic mutations and the latter by anti-phospholipid syndrome, acquired hyperhomocysteinemia, or other hypercoagulable diseases.

Anti-phospholipid syndrome is a group of noninflammatory autoimmune disorders caused by anti-phospholipid antibodies. This syndrome is characterized by thrombosis, recurrent pregnancy failure, and thrombocytopenia. Anti-cardiolipin antibody (ACA) is most closely associated with RSAs [6]. The primary target protein for the ACA is *β*2-GPI [7], and ACA can cause early miscarriage and mid- and late-term stillbirth in pregnancy by mediating the PI3K-Akt pathway. However, the exact mechanism of action is unclear. Previous studies have focused on abnormal immune homeostasis [8], thrombosis [9], trophoblast damage [10, 11], inflammation, and complement activation [12]. The incidence of ACA-positive recurrent miscarriage is approximately 20%, which has been increasing in the past few years [13], thereby adversely affecting the physical and mental health of patients.

The Bushen Huoxue (BSHX) recipe is an effective prescription based on the protocol of the Anhui Provincial Hospital of Traditional Chinese Medicine and has a significant effect on protecting the fetus. Our team's preliminary clinical studies show that the BSHX recipe combined with aspirin can significantly mitigate PTS in spontaneous abortion, leading to a pregnancy success rate of 90% [14]. For the treatment of recurrent abortions and PTS, the efficacy of this prescription is better than that of Western medicines in improving pregnancy outcomes and coagulation-related indicators. In addition, the rates of live and premature births were higher in the treatment group than in the control group [15]. The recipe focuses on the core pathogenesis of kidney deficiency and blood stasis. It is effective in treating PTS of RSA to protect the fetus [16]. This recipe enhances the functions of kidneys, nourishes the innate system, and strengthens the spleen to benefit the qi and blood to nourish the fetus. A previous study of this drug for the treatment of PTS in humans showed that this approach combined with aspirin significantly alleviated PTS in spontaneous abortions and improved coagulation function and the pregnancy success rate of patients [17]. The present study was conducted to investigate the molecular mechanism underlying the use of BSHX in the treatment of ACA-positive recurrent abortion with a prethrombotic state based on the PI3K-Akt signaling pathway and metabolomics to provide a theoretical basis and guidance for the optimal use of the drug in clinical practice.

## 2. Materials and Reagents

### 2.1. Instruments

The instruments used in this study included a QExac-tive™ HF-X mass spectrometer, Millipore Direct-Q3 Advantage ultrapure water system (Millipore, Bedford, MD, USA), and a BX43 biological inverted microscope (Olympus, Tokyo, Japan).

### 2.2. Medicinal Materials and Reagents

BSHX granules (Anhui Provincial Hospital of Traditional Chinese Medicine) consisted of 20 g of *Semen Cuscutae*, 15 g of *Herba Taxilli*, 15 g of *Radix Dipsaci*, 20 g of *Radix Codonopsis*, 15 g of *Rhizoma Atractylodis Macrocephalae*, 10 g of *Radix Angelicae Sinensis*, 10 g of *Radix Salviae Miltiorrhizae*, and 6 g of *Rhizoma Ligustici Chuanxiong*. Aspirin-enteric-coated tablets (batch no.: 190528206) were purchased from Shiyao group, Ouyi Pharmaceutical Co. Ltd., Shijiazhuang, China. Interleukin-6 (IL-6; batch no. 20201012-20188A; Shanghai Enzyme-linked Biotechnology Co. Ltd.) and anti-cardiolipin antibody (ACA; batch no. 20201012-22047A; Shanghai Enzyme-linked Biotechnology Co. Ltd.) test kits were purchased from the Shanghai Enzyme Link Biotechnology Co. (Shanghai, China).

Acetonitrile and formic acid were chromatographically pure (Merck, Darmstadt, Germany). Ultrapure water was generated by the Milli-Q system (Millipore).

### 2.3. Animal Husbandry

All experimental protocols were approved by the Animal Experimentation Ethics Committee of the Nanjing University of Chinese Medicine, China (no. 201912A001). CBA/J mice (90 females, unborn, 6-7 weeks old), DBA/2 (35 males, 6-7 weeks old), and BALB/c (10 males, 6-7 weeks old) were purchased from the Beijing Huafukang Biotechnology Co. (no. scxk2019-0008; Beijing, China) and housed at the Experimental Animal Centre of Nanjing University of Chinese Medicine. The temperature was maintained at 22 ± 2°C, relative humidity at 60 ± 2%, and light/dark cycle at 12 h.

### 2.4. Recurrent Miscarriage Mouse Model

After 7 days of acclimatization, female CBA/J mice were mated with male DBA/2 and male BALB/c mice at a ratio of 2:1 to establish a recurrent abortion model (CBA/J x DBA/2) and a normal pregnancy model (CBA/J x BALB/c), respectively. This was a recessive, recurrent, and paternally specific DBA/2XCBA of the Clark classical recurrent miscarriage mouse model [18]. Female mice were examined early the following morning, and those with vaginal plugs were classified as pregnant. Those without vaginal plugs were stained for vaginal secretions. Pregnancy was determined by the presence of a large number of spermatozoa on the vaginal smear combined with a wide-open vaginal opening. The presence of both signified day 1 of pregnancy.

### 2.5. Grouping and Administration of Drugs

Human *β*2 glycoprotein I was dissolved in a sterile phosphate-buffered solution (PBS) to obtain a solution of human *β*2 glycoprotein I at a mass concentration of 400 *μ*g/mL. Seventy CBA/J female mice received intraperitoneal injections of 50 *μ*L of a 1:1 mixture of human *β*2GPI and complete Freund's adjuvant (CFA) on day 1 and 50 *μ*L of a 1:1 mixture of human *β*2GPI and incomplete Freund's adjuvant (IFA) on day 8 to enhance immunization, resulting in an ACA-positive recurrent miscarriage mouse model. Sixteen CBA/J female mice received intraperitoneal injections of 50 *μ*L of saline on days 1 and 8, resulting in a normal pregnant mouse model. On day 18, 70 ACA-positive recurrent miscarriage mice were mated with male DBA/2 mice at a ratio of 2:1. Of these mice, 50 became pregnant, which were divided into 5 groups of 10 mice each: BSHX high-dose group (BH, 2.52 g/kg), BSHX medium-dose group (BM, 1.26 g/kg), BSHX low-dose group (BL, 0.63 g/kg), model group (M, distilled water), and aspirin enteric-coated tablets group (A, 3.25 mg/kg). Additionally, 16 CBA/J female mice in a normal pregnancy model were mated with male BALB/c mice, at a ratio of 2:1. Of these mice, 10 became pregnant, which were used as the blank control group (C). Each group was administered the corresponding drug by gavage on days 1–15, with groups M and C receiving the same volume of distilled water by gavage.

### 2.6. Sample Collection

Urine was collected from female mice 15 d after dosing the corresponding drug. Blood was collected from the orbits of female mice 24 h after the model was established and after 16 days of gestation for examination of the relevant indexes. Uterine tissues, including placenta and meconium, were also collected.

### 2.7. Testing Indicators

#### 2.7.1. Stillbirth and Abortion Rates

Stillbirth and abortion rates were calculated using the trypan blue stain method. A 4% tepantolam stock was prepared, diluted to 0.4% with PBS, and injected into the tail vein of the mice. After anesthesia, 4% trypan blue liquor was prepared, diluted to 0.4% with PBS, injected into the tail vein of mice, and then observed for 30 min. The mice were sacrificed by breaking the neck, and the embryos were observed to have a violet-blue stain at the embryonic implantation site. The violet-blue stain at the implantation site was also visible in the absence of normally developing embryos. This method was used to determine the total number of pregnancies; embryos with colored spots but no normal development were considered stillborn.

Stillbirth rate (%) was calculated as the number of stillbirths/number of stained fetuses at term × 100%.

#### 2.7.2. Histopathological Examination of the Uterine Metaplasm

The removed uterine metaplastic tissue specimens were fixed in a 4% paraformaldehyde solution for 24 h, rinsed, dehydrated, treated to appear transparent, wax-impregnated, embedded, and cut into 4-*μ*m-thick continuous transverse sections. This procedure was followed by baking the slides with wax slices in a drying oven at 75°C for 30 min. The slides were dewaxed in xylene and ethanol, rehydrated, stained with hematoxylin and eosin (HE), and sealed with neutral gum. HE was used to stain and observe the vascular and cellular morphology of uterine tissue of RSA mice under a light microscope.

#### 2.7.3. Measurement of the ACA, IL-6, P, and E2 Indicators

Blood was obtained from the orbits of mice, left for 2 h, and centrifuged at 4°C and 3,500 rpm/min for 10 min to extract the serum. The abovementioned indexes were tested according to the kit manufacturer's instructions.

#### 2.7.4. Data Processing

SPSS software (version 22.0; IBM Corp., Armonk, NY, USA) was used for the statistical analysis. The results are expressed as mean ± standard deviation ($\bar{x}$ ± *s*), with *P* < 0.05 indicating statistical significance.

### 2.8. Metabolomic Analysis

#### 2.8.1. Sample Preparation

Uterine tissues frozen at −80°C were thawed on ice, and samples of 100 mg were weighed, cut, and placed in a 2 mL centrifuge tube. A total of 300 *μ*L of acetonitrile was added, homogenized in a tissue grinder for 1.5 min, and centrifuged at 4°C for 10 min at 13,000 r/min. Then, the supernatant was extracted and centrifuged at 13,000 rpm/min for 10 min, followed by UHPLC-MS/MS analysis [17].

The urine samples were immediately centrifuged (3,000 rpm/min for 10 min), and the supernatant was stored in a refrigerator at −80°C. The urine sample was thawed at room temperature, and 400 *μ*L of urine was placed in a new centrifuge tube; 100 *μ*L of methanol was added; and the mixture was vortexed for 30 s. Then, the mixture was centrifuged (13,000 rpm/min for 10 min), and the supernatant was collected for UHPLC-MS/MS analysis [19].

#### 2.8.2. Chromatographic Conditions for Metabolomics

The chromatographic conditions consisted of the following. A Thermo SyncronisC_18_ column (2.1 mm × 100 mm, 1.7 *μ*m) andmobile phase is 0.1% formic acid water (A) - acetonitrile (B). The uterine tissue gradient elution conditions were 0–3.0 min, 5–45% B; 3.0–13.0 min, 45–95% B; 13.0–14.0 min, 95–5% B; and 14.0–14.2 min, 95-5% B. The urine samples elution conditions were 0–8.0 min, 5–30% B; 8.0–11.0 min, 30–70% B; 11.0–13.0 min, 70–95% B; 13.0–14.0 min, 95% B; and 14.0–14.2 min, 95–5% B.

The mass spectrometry conditions were as follows. Using an ESI ion source, a mass scan range m/z 100–1,000, capillary and cone voltages is 3.0 kV and 30 V, respectively. The cone and dissolvent gas flow rates were 50 L h^−1^ and 600 L h^−1^, respectively. The dissolvent gas temperature was 350°C, and the ion source temperature was 120°C with a scan time of 0.3 s and an interval scan time of 0.02. The exact mass was determined using a leucine-enkephalin (ESI^+^: 556.2771 *m/z* and ESI^−^: 555.2615 *m/z*) solution as a lock-in mass solution.

#### 2.8.3. Metabolomics Data Analysis

Data were analyzed using the MasslynxTM v4.2 workstation for noise removal, overlapping peak resolution, peak alignment, peak matching, and normalization. The data tables were analyzed to create a list of data in positive and negative ion modes according to the retention time, the mass-to-charge ratio (*m/z*), and normalized peak area for each peak region. Principal component analysis and orthogonal partial least squares discriminant analysis (OPLS-DA) were used to select variables with VIP >1 and |*P*| ≥ 0.05 as potential biomarkers [20]. SPSS software was used to test whether the relative peak areas of the biomarkers were significantly different between the two groups using the Student's *t*-test. The $\bar{x}$ ±*s* and the differential metabolites were screened for statistical significance (*P* < 0.05). Biomarker identification and metabolic pathway analysis were performed using the HMDB (https://www.hmdb.ca/) database and Metabo Analyst (https://www.metaboanalyst.ca/).

#### 2.8.4. PI3K/AKT Pathway-Related Proteins (PI3K, p-PI3K, AKT, and p-AKT) Assay

Uterine tissues were analyzed using a lysis solution, and the protein concentration of each protein sample was determined using a bicinchoninic acid assay (BCA) protein quantification kit. The average absorbance of each group of proteins was completed according to the spectrophotometer absorbance. The standard curve was plotted, and the regression equation was calculated. The extracted protein samples were denatured and subjected to gel concentration and gel separation electrophoresis. Polyvinylidene fluoride (PVDF) membranes of the same size as the gel samples were cut and placed on the transfer device, a glass stick was rolled to remove all bubbles, and the membrane was transferred to the PVDF membranes at 350 mA for 70 min. After transfer to the PVDF membranes, the protein membrane was immediately placed in 1×TBST and rinsed for 1 min. The membrane transfer liquid on the PVDF membrane was then washed, and the sealing liquid (5% skim milk powder dissolved in 1×TBST 50 mL) was added and shaken slowly for 1 h on a shaker. The corresponding primary antibodies (PI3 K, p-PI3 K, AKT, and p-AKT) were separately added for incubation. The membranes were transferred into a horseradish peroxidase (HRP) labeled goat anti-rabbit IgG antibody (1:1,000 dilution) for secondary antibody incubation, exposed after ECL development, scanned for protein results using a Biorad imaging system, and quantified using imaging software.

## 3. Results

### 3.1. Stillbirth and Abortion Rates

Compared with the normal group, the embryo absorption rate was significantly higher in the model group (*P* < 0.001). In addition, compared with the model group, the embryo absorption rate was significantly lower in the high-dose, medium-dose, and aspirin groups of the BSHX (*P* < 0.001; Table 1).

### 3.2. Histopathological Observation of Mouse Uterine Metaplasia

**Figure 1:**
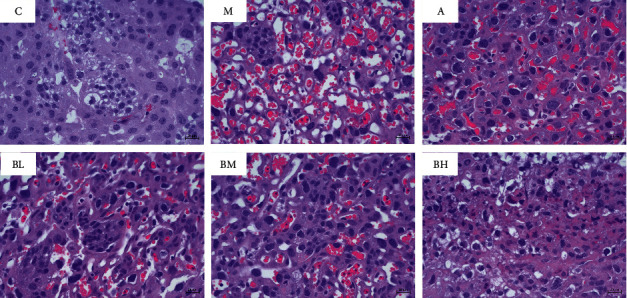
Pathological changes in the liver tissue (400×). C: control group; M: model group; A: aspirin group; BL: low-dose BSHX group; BM: medium-dose BSHX group; and BH, high-dose BSHX group.

The histopathological findings of the mouse uterine metaplasm (Figure 1) showed that the normal group had normal glandular development with a high number of glands, regular cell morphology, clear staining of the nucleus, and abundant cytoplasm. In addition, the normal group had a higher number of blood vessels with an intact wall and no stasis on the wall of the tube. The model group showed incomplete vessel walls, apoptotic cells, and obvious cellular vacuole-like changes with stasis in the vessels and vacuole-like structures in the placenta. In the aspirin group, the number of blood vessels was reduced, and the morphology was normal. However, vacuolated cells and apoptotic cells were evident. In addition, there was bruising in the blood vessels, and vacuolated structures were visible in the placenta. The number of blood vessels was normalized in the BSHX high-dose group. Additionally, the blood vessel wall was intact, apoptotic cells; vacuole-like changes were significantly reduced; intravascular stasis was significantly improved; and vacuole-like structures in the placenta were rare. In the BSHX middle-dose group, the number of blood vessels was normal, and the vessel wall was intact, but a small number of apoptotic cells and vacuolated cells were observed. In the BSHX low-dose group, the number of blood vessels was significantly reduced; the vessel wall was incomplete; apoptotic cells and vacuolated cells were observed; and there was blood stasis in the vessels and vacuolated structures in the placenta. These results suggested that the high-dose, medium-dose, and low-dose BSHX treatments improved the morphology of uterine tissue, with the most significant improvement in the high-dose group.

### 3.3. Measurement of the Relevant Indicators

**Figure 2:**
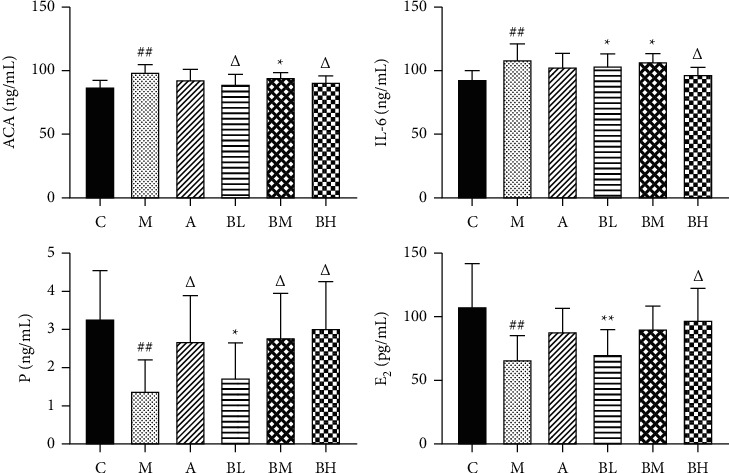
Levels of ACA, IL-6, P, and E2 in the serum of each group. C: control group; M: model group; A: aspirin group; BL: low-dose BSHX group; BM: medium-dose BSHX group; and BH” high-dose BSHX group. ##*P* < 0.01, model group versus control group; ^*∗*^*P* < 0.05, treatment groups versus control group; and ^Δ^*P* < 0.05, treatment groups versus model group.

Compared with the blank group, ACA and IL-6 levels were significantly increased in the serum of the model group (*P* < 0.01), and their levels were reduced by treatment with aspirin enteric-coated dissolved tablets and the BSHX recipe (Figure 2). The low- and high-dose groups of the BSHX recipe significantly reduced ACA to normal levels, and the high-dose group significantly reduced IL-6 levels (*P* < 0.05). Compared with the blank group, serum progesterone and estradiol levels were significantly decreased in the model mice. In addition, their levels were increased by treatment with the BSHX recipe and aspirin enteric-coated tablets. The levels were significantly increased in the medium- and high-dose groups, as compared with the aspirin enteric tablets group and the low-dose group.

### 3.4. Metabolomic Analysis

#### 3.4.1. Multivariate Analysis between the Normal and Model Groups

**Figure 3:**
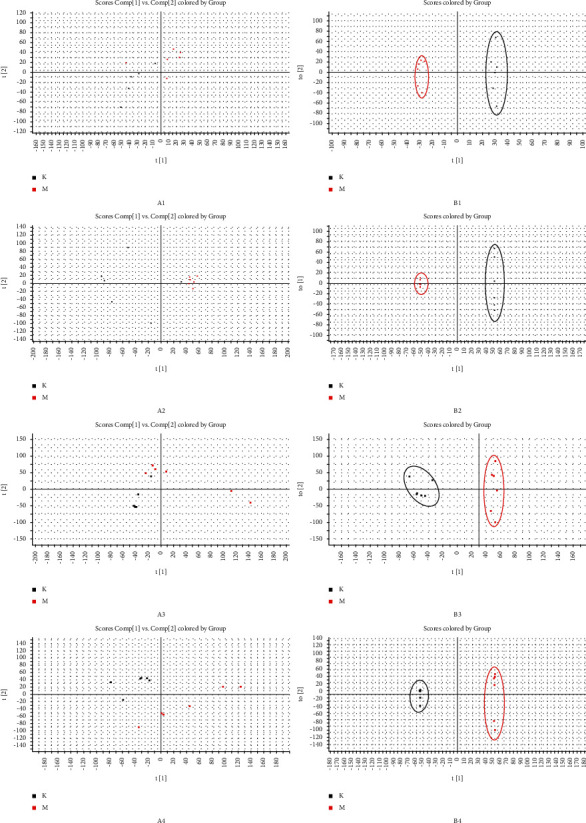
PCA score plots (a) and OPLS-DA score plots (b) for the uterus (labels 1 and 2) and urine samples (labels 3 and 4). Labels 1 and 3 were obtained in negative ion mode, and labels 2 and 4 were obtained in positive ion mode.

To examine the overall changes in the metabolic levels in the aborted mice, principal component analysis and OPLS-DA were used to analyze the metabolomic data of the normal and model biological samples. Urine and uterine tissue samples from the model group were significantly different from the corresponding samples from the normal group, indicating that the metabolic levels in the mice of the abortion model were significantly abnormal (Figure 3).

#### 3.4.2. Analysis and Identification of Potential Biomarkers

**Figure 4:**
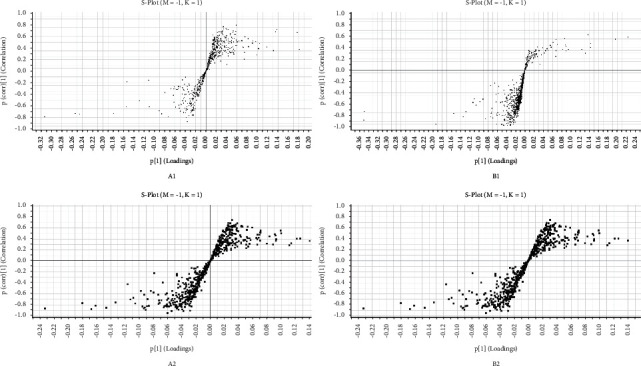
*S*-plot for the uterus and urine samples. (a) negative ion mode and (b) positive ion mode.

Differential variables between the two groups were screened for potential biomarkers using *S*-plot score plots in the OPLS-DA analysis mode as well as the VIP values. As shown in Figure 4, in the *S*-plot, the points distant from the metabolites were the major ion clusters, indicating a greater contribution to the differentiation of the sample categories and a higher likelihood of the usefulness as potential biomarkers. Therefore, variables with VIP >1 and |*P*| ≥ 0.05 were extracted as potential markers. Compounds with significant differences (*P* < 0.05) on a *t*-test were imported into the HMDB database. The HMDB database was searched, and 30 potential biomarkers were screened for abnormalities in urine and uterine tissue samples (Table 2).

#### 3.4.3. Evaluation of the Intervention Effect of the BSHX Recipe on Mice with Recurrent Miscarriage

**Figure 5:**
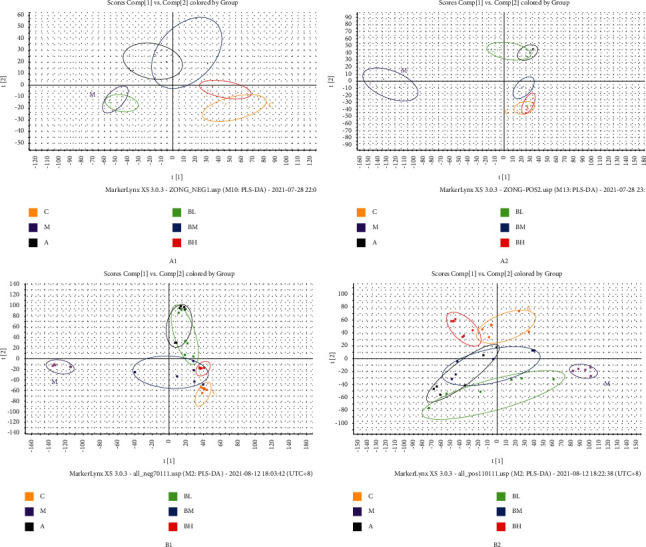
PLS-DA score plots for the uterus (a) and urine samples (b). Label 1 was obtained in a negative ion mode, and label 2 was obtained in a positive ion mode. C: control group; M: model group; A: aspirin group; BL: low-dose BSHX group; BM: medium-dose BSHX group; and BH: high-dose BSHX group.

The UPLC/Q-TOF/MS data of the blank group, model group, aspirin group, and high-, medium-, and low-dose groups of the BSHX recipe were discriminatively analyzed using an OPLS-DA. As shown in Figure 5, uterine and urine samples of mice in the administration groups were clearly distinguished from those from the model group, indicating that the metabolic abnormalities in mice with RSA were improved after treatment with BSHX.

**Figure 6:**
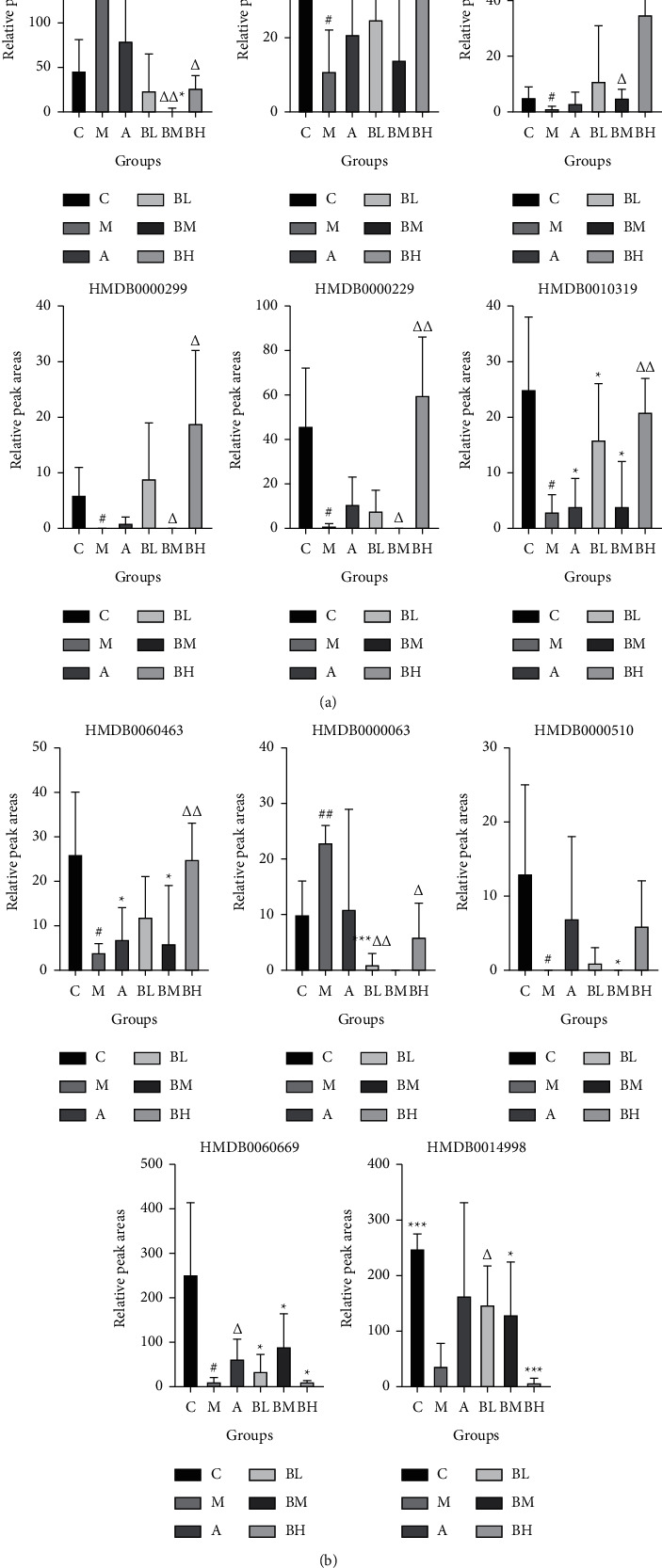
Relative peak area of potential biomarkers identified in the urine (*###P* < 0.001, *##P* < 0.01, and *#P* < 0.05, model group vs. control group; ^*∗∗∗*^*P* < 0.001, ^*∗∗*^*P* < 0.01, and ^*∗*^*P* < 0.05, treatment groups vs. control group; ^ΔΔΔ^*P* < 0.001, ^ΔΔ^*P* < 0.01, and ^ΔΔ^*P* < 0.05, treatment groups vs. model group).

**Figure 7:**
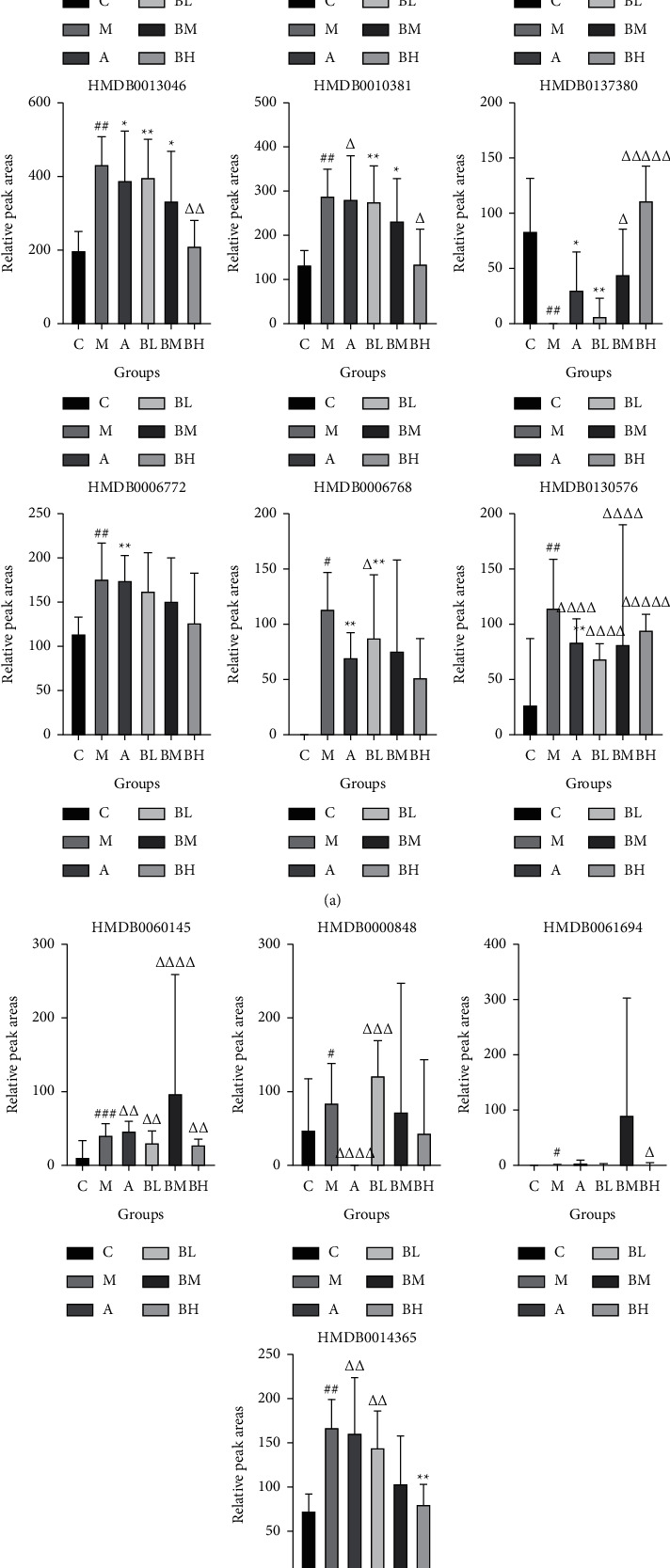
Relative peak area of potential biomarkers identified in the uterus (*###P* < 0.001, *##P* < 0.01, and *#P* < 0.05, model group vs. control group; ^*∗∗∗*^*P* < 0.001, ^*∗∗*^*P* < 0.01, and ^*∗*^*P* < 0.05, treatment groups vs. control group; ^ΔΔΔ^*P* < 0.001, ^ΔΔ^*P* < 0.01, and ^ΔΔ^*P* < 0.05, treatment groups vs. model group).

Among the identified potential biomarkers, relative levels of the markers in the model group showed an increasing trend; however, some were downregulated compared to the blank group. After administration of high-, medium-, and low-dose BSHX, the levels of 24 potential biomarkers were regulated back toward the blank group, indicating that the BSHX recipe had a modulatory effect on the metabolic status of mice with RSA. The relative levels of the potential biomarkers in each group of samples (expressed as peak areas derived from the UPLC-Q-TOF/MS analysis) are shown in Figures 6 and 7.

#### 3.4.4. Analysis of the Metabolic Pathways

**Figure 8:**
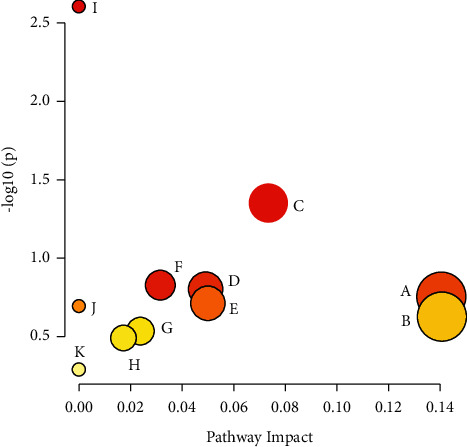
Metabolic pathways. A: pentose and glucuronate interconversions; B: lysine degradation; C: steroid hormone biosynthesis; D: histidine metabolism; and E: citrate cycle (TCA cycle).

The results of the 24 potential biomarkers entered into the Metabo Analyst 4.0 online analysis database for pathway enrichment analysis to identify the involved metabolic pathways are shown in Figure 8. Among these, the pentose and glucuronate interconversions, lysine degradation, and steroid hormone biosynthesis were found to be the main metabolic pathways involved.

### 3.5. PI3K/AKT Pathway-Related Protein (AKT, p-AKT, PI3K, and p-PI3K) Assays

**Figure 9:**
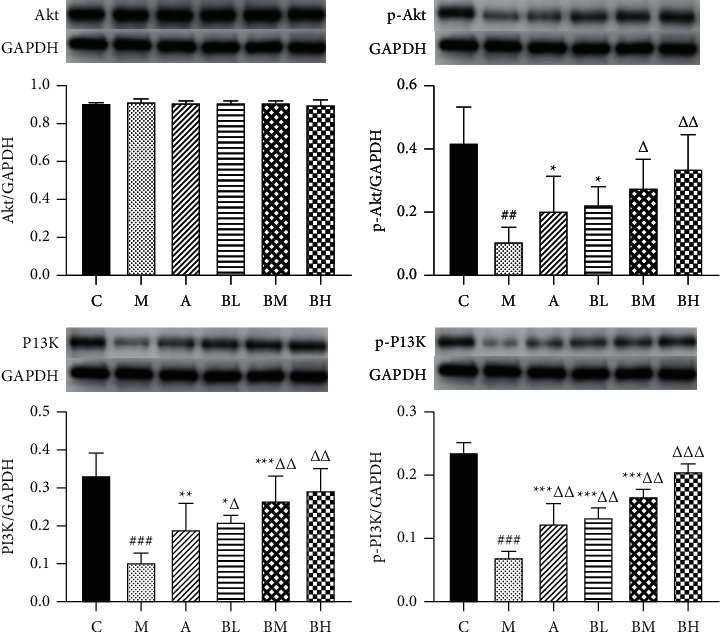
Comparison of the relative expression levels of the AKT, p-AKT, PI3K, and p-PI3K proteins in the placenta of pregnant mice in each group. C: control group; M: model group; A: aspirin group; BL: low-dose BSHX group; BM: medium-dose BSHX group; and BH: high-dose BSHX group. ###*P* < 0.001, model group versus control group; ^*∗∗∗*^*P* < 0.001, ^*∗∗*^*P* < 0.01, and ^*∗*^*P* < 0.05, treatment groups versus control group; and ^ΔΔ^*P* < 0.01 and ^ΔΔ^*P* < 0.05, treatment groups versus model group.

The expression levels of the PI3K/AKT pathway-related proteins in the mice placental tissues were analyzed in grey scale (Figure 9). Compared with the normal group, expression levels of the *p*-AKT, PI3K, and *p*-PI3K proteins in mice's placental tissues of the model group were significantly decreased. Compared with the model group, the expression levels of the *p*-AKT, PI3K, and *p*-PI3K proteins in the BSHX-treated groups were significantly increased in a dose-dependent manner. In addition, there was no significant difference in the expression level of the AKT protein in the placental tissues of mice in each group.

## 4. Discussion

Recurrent miscarriage is known as “slippage” in Chinese medicine. According to the Chinese medicine theory, two mechanisms may lead to slippage: damage to the mother's body and an unhealthy fetus. The etiology of RSA is complex and includes physical, human, biological, prethrombotic, and immune factors. Autoimmune factors, such as anti-phospholipid antibodies, are important factors leading to RSA. ACA is the autoantibody most closely related to the occurrence of RSA. Recent studies have shown that it directly acts on placental vascular endothelial phospholipids, inhibits PG12 secretion, stimulates the release of arachidonic acid, and accelerates platelet aggregation and vasoconstriction, thus causing placental thrombosis and infarction, resulting in unexplained recurrent pregnancy loss due to hypoxia [21, 22]. Additionally, ACA inhibits trophoblast function and decreases the production of syncytial trophoblasts, resulting in insufficient secretion of placental hormones to maintain pregnancy, leading to impaired blastocyst implantation and miscarriage due to meconium damage. ACA also affects thrombomodulin, resulting in decreased production of protein C and protein S and decreased fibrinogen activation. Finally, ACA can cause *β*2-GP-1 to bind to ACA and inhibit the anti-coagulant activity of *β*2-GP-1. The results of this study showed that the expression of serum ACA was significantly reduced after treatment with the kidney tonic and blood activation formula. Therefore, the mechanism of action of the kidney tonic and blood activation formula for RSA may be related to the abovementioned pathways.

The PI3K-AKT signaling pathway is a classical signaling pathway for intracellular proliferation and apoptosis; it regulates various cellular functions, including cell growth, angiogenesis, and apoptosis. Therefore, ACA and the PI3K/AKT signaling pathway are closely related in terms of their cellular and vascular effects. Blocking the PI3K-AKT signaling pathway can inhibit the growth and invasion of human trophoblast cells, thereby affecting placental formation and maintenance of early pregnancy in humans [23]. However, activating the PI3K-AKT signaling pathway upregulates the secretion of vascular endothelial growth factors in trophoblast cells and regulates villi trophoblast function [24].

During early embryonic development, cytotrophoblasts are involved in cell invasion and proliferation, whereas syncytial trophoblasts have endocrine functions and produce a variety of pregnancy-related cytokines and hormones, such as progesterone, estradiol, and interleukins [25, 26]. Progesterone is primarily secreted by the ovarian corpus luteum and plays an important role in fertilized egg implantation and early pregnancy maintenance. It has various effects such as inhibiting maternal immune rejection, regulating membrane permeability of uterine smooth muscle cells, regulating invasion and migration ability of trophoblast cells, reducing the sensitivity of the uterus to contractile hormones, and inhibiting the contraction of uterine smooth muscle and anti-phospholipid to promote the implantation of fertilized eggs and normal development in the uterus [27–29].

The results of this study showed that the expression levels of p-AKT, PI3K, and p-PI3K proteins in the endometrial tissues of ACA-positive mice with RSA were significantly increased after BSHX treatment. In addition, metabolomics showed that BSHX promoted steroid hormone biosynthesis and significantly increased the levels of estrogen and progesterone hormones *in vivo* while activating the PI3K/AKT signaling pathway. BSHX also activates the PI3K/AKT signaling pathway, inhibits apoptosis of the chorionic trophoblast, and increases the number of normal functioning trophoblast cells in the body, especially the number of syncytial trophoblast cells. This further increases the expression level of progesterone in the body, thus maintaining egg fertilization and normal development. In addition, the recipe significantly improved the histopathological morphology of the endometrium, as evidenced by the normalization of the number of blood vessels, integrity of the vessel wall, and reduction in intravascular stasis. This suggests that the recipe can interfere with the PI3K-Akt signaling pathway, participate in trophoblast function, and promote angiogenesis in uterine tissues, thus exerting an anti-fetal effect.

The BSHX recipe is a Chinese herbal prescription composed of dodder, mulberry parasite, Dipsacus, *Radix angelicae*, *Atractylodes macrocephala*, *Angelica sinensis, Salvia miltiorrhiza,* and *Ligusticum chuanxiong*. Currently, anti-coagulant therapy is mainly used for patients with recurrent abortions and positive anti-phospholipid antibodies. Previous studies have shown that *Salvia miltiorrhiza*, *Angelica sinensis*, and *Ligusticum chuanxiong* improve blood circulation [30–32]. In addition, *Salvia miltiorrhiza* and *Radix Angelicae sinensis* prevent thrombosis [33, 34], whereas *Salvia miltiorrhiza* and Codonopsispilosula improve the microcirculation [30, 35, 36] and regulate immunity [37–39]. In addition, the tonic recipe also intervenes in the metabolic pathways of the interconversion of pentose and glucuronide, lysine degradation, and histidine metabolism in the body. The interconversion pathway of pentose and glucuronide is related to the choking reaction. Glucuronide has a detoxifying effect, which is related to the clearing of many exogenous and endogenous compounds in the body. It plays an important role in the metabolic clearing of poisons, endogenous hormones, and bile acids in the body. However, histidine and lysine are the basic components of proteins and are involved in many physiological processes, making them indispensable for living organisms. Importantly, histidine participates in the protein composition of an organism, regulation of the nervous system, and has a certain protective effect on the organism [28, 40, 41].

Early clinical practice showed that BSHX recipe can reduce PTS in spontaneous abortion. However, there are many causes of PTS. This experimental study used an animal model of prethrombotic state of ACA-positive recurrent abortion, which is highly targeted. The relationship between BSHX and the PI3K-AKT pathway was determined at an early stage, but pathway inhibitors or gene knockout should be used to determine whether the PI3K-AKT pathway is involved in the treatment process. Furthermore, we should investigate the mechanism of action of BSHX at the late stage to provide a basis for the clinical use of traditional Chinese medicine preparations during pregnancy.

## 5. Conclusion

The BSHX recipe upregulated the levels of *p*-AKT, PI3K, and *p*-PI3K proteins. Under the BSHX recipe, PI3K may be phosphorylated into P-PI3K, leading to the activation of the main downstream effector Akt, which in turn activates the PI3K-AKT signaling pathway and increases *p*-AKT levels. The recipe reduces serum ACA, thereby reducing the platelets, vasoconstriction rate, and effect on thrombomodulin, as well as improving the trophoblast and coagulation functions. Furthermore, the recipe can reduce the IL-6 levels and increase progesterone and estradiol levels, which play an important role in regulating the maternal and fetal immune tolerance to maintain a normal pregnancy. The recipe can also regulate the *in vivo* metabolic pathways of pentose and glucuronide interconversion, lysine degradation, and steroid hormone biosynthesis. Thus, the metabolic disorders in mice were improved and the levels of estrogen and progesterone were increased. These effects promoted embryonic development and reduced the rate of embryo resorption in the treatment of ACA-positive recurrent miscarriages.

## Figures and Tables

**Table 1:** Comparison of the embryonic mortality of each group (*n* = 10, $\bar{x}$ ± s).

Group	Number of stillbirths	Number of beds	Stillbirth rate (%)
C	8	82	9.76
M	32	80	40.00^###^
A	13	74	17.56^ΔΔΔ^
BL	12	71	35.21
BM	14	76	18.42^ΔΔΔ^
BH	12	79	15.19^ΔΔΔ^

(1) Compared with the control group, ###*P* < 0.001; (2) compared with the model group, ^ΔΔΔ^*P* < 0.001. C: control group, M: model group, A: aspirin group, BL: low-dose BSHX group, BM: medium-dose BSHX group, and BH: high-dose BSHX group.

**Table 2:** Identification of the potential biomarkers.

NO.	Rt	HMDB	Metabolites	*m/z*	Formula	Content variance	Ion mode	Source
M1	8.86	HMDB0030357	N-Methylaspidospermatidine	280.4073	C_19_H_24_N_2_	↑	−	Uterus
M2	13.92	HMDB0033408	4-Methoxycinnamoyloleanolic acid methyl ester	630.8962	C_41_H_58_O_5_	↑	−	Uterus
M3	12.28	HMDB0011511	LysoPE(20:0/0:0)	509.6566	C_25_H_52_NO_7_P	↑	−	Uterus
M4	11.62	HMDB0060785	6-Hydroxyemedastine	318.4139	C_17_H_26_N_4_O_2_	↑	−	Uterus
M5	10.47	HMDB0013046	Psychosinesulfate	541.696	C_24_H_47_NO_10_S	↑	−	Uterus
M6	10.46	HMDB0010381	LysoPC(15:0/0:0)	541.657	C_28_H_48_NO_7_P	↑	−	Uterus
M7	10.28	HMDB0137380	(4E)-6-Hydroxy-1-(4-hydroxy-3-methoxyphenyl)dodec-4-en-3-one	320.429	C_19_H_28_O_4_	↓	−	Uterus
M8	9.54	HMDB0006772	Adrenosterone	300.3921	C^19^H^24^O^3^	↑	−	Uterus
M9	12.03	HMDB0000010	2-Methoxyestrone	300.3921	C^19^H^24^O^3^	↓	−	Uterus
M10	10.57	HMDB0006768	19-Oxoandrost-4-ene-3,17-dione	300.3921	C^19^H^24^O^3^	↑	−	Uterus
M11	11.22	HMDB0130576	3-Phenyl-1-[2,4,6-trihydroxy-3-(hydroxymethyl)-5-methylphenyl]prop-2-en-1-one	300.31	C_17_H_16_O_5_	↓	+	Uterus
M12	10.48	HMDB0060145	Alpha-tocotrienoxyl radical	423.6505	C_29_H_43_O_2_	↓	+	Uterus
M13	12.45	HMDB0000848	Stearoylcarnitine	428.677	C_25_H_50_NO_4_	↓	+	Uterus
M14	13.03	HMDB0061694	1-Oleoylglycerophosphoserine	523.604	C_24_H_46_NO_9_P	↓	+	Uterus
M15	9.66	HMDB0014365	Nelfinavir	567.782	C_32_H_45_N_3_O_4_S	↓	+	Uterus
M16	1.86	HMDB0000072	cis-Aconitic acid	174.1082	C_6_H_6_O_6_	↑	−	Urine
M17	2.56	HMDB0004063	Metanephrine	197.231	C_10_H_15_NO_3_	↓	−	Urine
M18	1.42	HMDB0060367	3-Carbamoyl-2-phenylpropionic acid	209.1986	C_10_H_11_NO_4_	↓	−	Urine
M19	2.22	HMDB0000194	Anserine	240.259	C_10_H_16_N_4_O_3_	↓	−	Urine
M20	3.12	HMDB0000299	Xanthosine	284.2255	C_10_H_12_N_4_O_6_	↓	−	Urine
M21	2.57	HMDB0000229	Nicotinamide ribotide	335.2271	C_11_H_16_N_2_O_8_P	↓	−	Urine
M22	2.25	HMDB0010319	Indoxyl glucuronide	309.2714	C_14_H_15_NO_7_	↓	−	Urine
M23	1.8	HMDB0060463	Citalopram propionic acid	311.3071	C_18_H_14_FNO_3_	↓	−	Urine
M24	3.46	HMDB0000063	Cortisol	362.4599	C^21^H^30^O^5^	↑	−	Urine
M25	2.39	HMDB0002017	1-Phenylethylamine	121.1796	C_8_H_11_ N	↓	+	Urine
M26	2.42	HMDB0032568	2′-Hydroxyacetophenone	136.1479	C_8_H_8_O_2_	↓	+	Urine
M27	2.44	HMDB0000510	Aminoadipic acid	161.1558	C_6_H_11_NO_4_	↓	+	Urine
M28	3.12	HMDB0000017	4-Pyridoxic acid	183.1614	C_8_H_9_NO_4_	↓	+	Urine
M29	3.03	HMDB0060669	p-Hydroxyfelbamate	254.2393	C_11_H_14_N_2_O_5_	↓	+	Urine
M30	3.52	HMDB0014998	Prednisolone	360.444	C^21^H^28^O^5^	↓	+	Urine

## Data Availability

The data used to support the findings of this study are included within the article.
